# Letter from Dr. Barrett

**Published:** 1899-04

**Authors:** 


### Domestic Correspondence.

### LETTER FROM DR. BARRETT.

TO THE EDITOR:

SIR,—The following is only one of a number of similar letters already received by the Dental Department of the University of Buffalo, all of which have received like answers. It shows how widely the fraudulent college has sold its degrees, and the use which is being made of the counterfeit diplomas. Suit has been commenced against the institution named, and a decree against it has been obtained, but as it has *eight* different charters granted by the authorities of the State of Illinois, it will take time to close it up altogether. It is hoped that before the annual meeting of the National Association of Dental Faculties in August next, such legislation will have been obtained as will put a stop to the infamous diploma traffic by the dozen different irregular colleges of Chicago and the West.

BUFFALO, N. Y., February 10, 1899.

"BUFFALO DENTAL DEPARTMENT:

"Being a graduate of the ——— Medical College of Chicago, Ill., I take the pleasure in asking you how many years you will allow me in your college. I have a diploma of D.D.S., and wish to graduate from your college. If you will allow me any length of time I will start next term. Thank you for your kindness.

"Truly yours,

"O. B."

(*Answer.*)

"O. B.:

"SIR,—Your letter is at hand, and I must say that your assurance astounds even me, accustomed as I am to such insulting propositions. If you hold a diploma from the college named, you bought it, and have no more knowledge of medicine than you had before the bargain. You know it to be a fraudulent concern, and I know that the man who will try to profit by such a transparent fraud is no better than the miserable concern whose diploma he attempts to use. The man who knowingly tries to put a counterfeit in circulation is no better than the original counterfeiter. Your diploma is a badge of disgrace, and this college would consider itself dishonored by the presence of such a man among its students. Under no circumstances would we receive you, even though you should offer us ten times the usual fees. You would contaminate the very atmosphere of the college.

"Furthermore, let me inform you that the honorable profession of dentistry is taking steps to prevent the intrusion of men who attempt to obtain admission by fraud. We have commenced suit against this so-called 'Medical College,' which has advertised its wares so extensively, and have substantially a decree against it. We shall have full lists of the 'graduates' of the fraudulent concern, and they will be published, so that they may be excluded from all decent colleges. Not only will you be unable to profit by your intended fraud, but you will be severely punished if you attempt to make use of it. In this State it is a penal offence to attempt to use such a diploma, and if you even show it to others I promise that you shall see the inside of a jail.

"All this is of course based upon your own statement, which I understand to be that you hold a diploma from a fraudulent college and are attempting to use it for your own benefit. If this is not the case, then what I say has no application.

"Yours as above,

"W. C. BARRETT,

"*Dean.*"

